# Crystal structures of multicopper oxidase CueO G304K mutant: structural basis of the increased laccase activity

**DOI:** 10.1038/s41598-018-32446-7

**Published:** 2018-09-24

**Authors:** Hanqian Wang, Xiaoqing Liu, Jintong Zhao, Qingxia Yue, Yuhua Yan, Zengqiang Gao, Yuhui Dong, Zhiyong Zhang, Yunliu Fan, Jian Tian, Ningfeng Wu, Yong Gong

**Affiliations:** 1 Multi-discipline Center, Institute of High Energy Physics, Chinese Academy of Sciences, Beijing, 100049 China; 2 Biotechnology Research Institute, Chinese Academy of Agricultural Sciences, Beijing, 100081 China; 3 Institute of Physical Science and Information Technology, Anhui University, Hefei, Anhui 230601 China; 4 Beijing Synchrotron Radiation Facility, Institute of High Energy Physics, Chinese Academy of Sciences, Beijing, 100049 China; 5 CAS Key Laboratory for Biomedical Effects of Nanomaterials and Nanosafety, CAS Key Laboratory of Nuclear Radiation and Nuclear Energy Technology, Institute of High Energy Physics, Chinese Academy of Sciences, Beijing, 100049 China

## Abstract

The multicopper oxidase CueO is involved in copper homeostasis and copper (Cu) tolerance in *Escherichia coli*. The laccase activity of CueO G304K mutant is higher than wild-type CueO. To explain this increase in activity, we solved the crystal structure of G304K mutant at 1.49 Å. Compared with wild-type CueO, the G304K mutant showed dramatic conformational changes in methionine-rich helix and the relative regulatory loop (R-loop). We further solved the structure of Cu-soaked enzyme, and found that the addition of Cu ions induced further conformational changes in the R-loop and methionine-rich helix as a result of the new Cu-binding sites on the enzyme’s surface. We propose a mechanism for the enhanced laccase activity of the G304K mutant, where movements of the R-loop combined with the changes of the methionine-rich region uncover the T1 Cu site allowing greater access of the substrate. Two of the G304K double mutants showed the enhanced or decreased laccase activity, providing further evidence for the interaction between the R-loop and the methionine-rich region. The cuprous oxidase activity of these mutants was about 20% that of wild-type CueO. These structural features of the G304K mutant provide clues for designing specific substrate-binding mutants in the biotechnological applications.

## Introduction

Multicopper oxidases (MCOs) are a large, widely distributed and diverse family of enzymes with various functions ranging from copper (Cu) and iron metabolism to polyphenol oxidation^1^. One important feature of MCOs is that a minimum of four Cu ions are arranged at two sites: the mononuclear type 1 Cu centre (T1), and the trinuclear Cu cluster (TNC) consisting of a type 2 Cu centre (T2) and a dinuclear type 3 centre (T3)^1,2^. Four single electron-transfer reactions from the T1 site to the TNC cluster are coupled to the oxidation of various substrates. Electrons are passed to dioxygen bound to the TNC to generate two water molecules.

Laccases are the largest subfamily of MCOs and widely distributed in diverse organisms. They have multiple functions, including lignin biosynthesis and wound healing in plants; lignin degradation, pigmentation, and pathogenesis in fungi; and pigment production in the endospore coat in some bacteria such as *Bacillus subtilis*^3^. Laccases can catalyse the oxidation of a broad spectrum of organic substrates such as polyphenols, diamines, and some inorganic compounds^4^. These enzymes have been received increasing attention because of their practical applications in the textile, food, and wood-processing industries and their possible uses in bioremediation^5^.

CueO, an MCOs from *Escherichia coli* (*E*. *coli*), together with CopA, a P-type ATPase, comprises a copper efflux (*cue*) system that resists copper stress under aerobic conditions^6,7^. Expression of both enzymes is stimulated by exogenous Cu ions via the cytosolic metalloregulatory protein CueR^8,9^. Previous analysis of the crystal structure of *E*. *coli* CueO has highlighted that it adopts a canonical architecture consisting of three similar cupredoxin-like domains linked by peptides. This architecture is shared by the other known laccase and ascorbate oxidase. Within domain III, a unique 42-residue methionine-rich region made up mainly of methionine-rich helix (thereafter, MR helix) may hinder the access of bulky organic substrates to the T1 Cu site^10^. This region near the T1 Cu position was later found to coordinate a labile fifth Cu atom, a novel feature of CueOs that differs from other MCOs^11^. Like other common laccases, CueO exhibits phenol oxidase activity with a broad range of substrates. However, this enzyme possesses an unique cuprous oxidase activity *in vitro*^6,12^. CueO can catalyze the oxidation of cuprous ion (Cu(I)) to the less toxic cupric ion (Cu(II)) *in vivo*^12^.

Recent structural and functional studies of engineered *E*. *coli* CueO have showed that removal of the methionine-rich helical region significantly decreased cuprous oxidase activity of the enzyme, but increased its phenol oxidase activity. These findings implied that the MR helix region confers the specific cuprous oxidase of CueO^13^. Djoko *et al*. provided compelling evidence that the higher affinity of the methionine-rich region for Cu (I) over Cu (II) explains why CueO functions solely as a cuprous oxidase but not phenol oxidase *in vivo*^14^. Recent analyses of the crystal structures of CueO bound to Cu (I) provided evidence that the methionine-rich region binds and oxides Cu (I)^15^.

Very recently, we characterized the *E*. *coli* CueO G304K mutant (hereafter, G304K) from the metagenome of the sludge in a chemical plant, and found that catalytic efficiency of the mutant towards an organic substrate in the presence of excess Cu (II) was 2.7-folds higher than that of the wild-type enzyme^16^. In the present work, we report the crystal structure of G304K at a resolution of 1.49 Å. We also report the structure of G304K in the presence of excess Cu ions at 2.20 Å. Compared with the wild-type enzyme, G304K showed dramatically conformational changes, with the excess Cu ions inducing the further conformation changes. Combined with the structural information, functional assays highlighted that the regulatory loop (hereafter, R-loop) may modulate the methionine-rich region to regulate the activity of the enzyme.

## Results

### Overall structure of G304K in the presence of excess Cu ions

Recently, we characterized G304K, which shows markedly increased the laccase activity compared with that of the wild-type enzyme^16^. To find out the reason for the increase in laccase activity, we determined the crystal structure of G304K at a resolution of 1.49 Å (Table 1).

**Table 1:** Data collection and refinement statistics.

	G304K mutant	G304K mutant (Cu-soaked)	
		Refinement Data Set	Data Set collected at the Cu absorption edge
Data collection	SSRF-BL17U	PF-BL1A	PF-BL5A
Wavelength (Å)	0.98	1.1	1.37
Space group	P2_1_	P2_1_	P2_1_
Cell dimensions			
*a*, *b*, *c* (Å)	62.3, 50.7, 66.2	62.6, 50.6, 66.6	62.4, 50.6, 66.6
α, β, γ (°)	90.0, 98.8, 90.0	90.0, 93.9, 90.0	90.0, 94.0, 90.0
Resolution (Å)	50–1.49 (1.52–1.49)^a^	50–2.20 (2.24–2.20)^a^	50–2.45 (2.49–2.45)^a^
*R*_merge_	0.040 (0.459)	0.046 (0.624)	0.078 (0.730)
〈*I*/σ(*I*)〉	26.9 (2.5)	21.9 (1.5)	32.6 (1.8)
Completeness (%)	98.6 (88.6)	99.7 (96.0)	99.7 (93.6)
Redundancy	3.6	6.4	7.3
Refinement			
Resolution (Å)	50–1.49	50–2.20	
No. reflections	65,673	21,166	
*R*_work_/*R*_free_	0.145/0.180	0.189/0.236	
No. atoms			
Protein	3,551	3,517	
Cu	3	7	
Water	512	133	
B-factors	19.8	49.0	
Rmsd bond length (Å)	0.005	0.005	
Rmsd bond angle (°)	0.9	0.7	
Ramachandran Plot			
Favoured (%)	97.2	96.7	
Allowed (%)	2.8	3.3	
Outliers (%)	0.0	0.0	

^a^The values in parenthesis mean those of the highest resolution shell.

**Figure 1:**
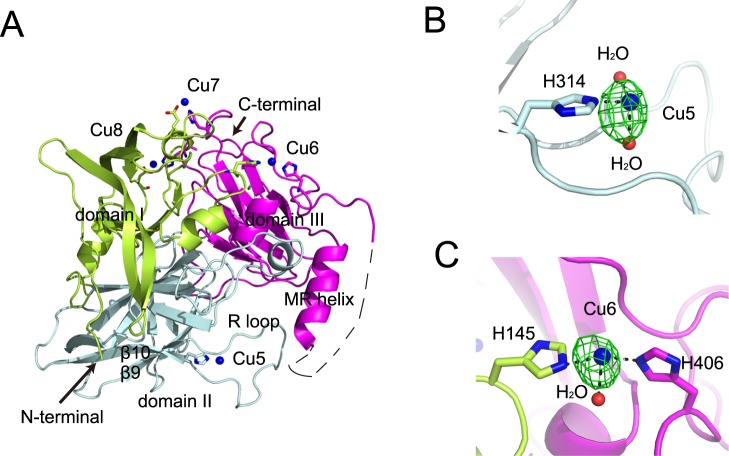
Overall structure of CueO G304K mutant in the presence of excess copper ions. (**A**) Overall structure of G304K mutant. Domain I-III is colored limon, palecyan, and magenta, respectively. (**B**) Residue H314 shown as sticks, and two water molecular indicated with red spheres, coordinate Cu5 (blue sphere). (**C**) Residues H145 and H406 shown as sticks, and one water molecular indicated with red sphere, coordinate Cu6 (blue sphere). In (**B** and **C**) the anomalous difference Fourier maps (green, contour at 4.5σ) are shown for Cu5 and Cu6.

As the maximum laccase activity of CueO is achieved only in the presence of excess Cu ions^10^, we soaked G304K crystals in Cu-containing solution and determined the complex structure at a resolution of 2.20 Å (Table 1). The resolved G304K structure is essentially identical to that of wild-type protein^10^ (PDB entry 1KV7). Similar to most of the reported crystal structures of CueO, the loop (residues 370–398) near T1 Cu atom is disordered in the G304K structure, suggesting that it is flexible (Fig. 1A). One of the most obvious features is the presence of several new Cu-binding sites only found on the surface of G304K structure after soaked with Cu ions. The presence of these extra Cu-binding sites was confirmed by the analysis of the anomalous difference Fourier map (Figs 1 and S1A,B). Among these Cu-binding sites, Cu5 and Cu6 were located around the MR helix (residues 357–369). Cu5 was coordinated by His314, located on the R-loop (residues 299–314), and two water moleculars (Fig. 1B). Cu6 was coordinated by His145, His405, and a water molecular (Fig. 1C). There were two other Cu ions found in the Cu-soaked G304K structure: Cu7 and Cu8. Cu7 was coordinated by His495, Glu110, and also found in Δα5–7 CueO structure^13^ (PDB entry 2YXW); whereas Cu8 was linked with His488, Asp132, and a water molecular (Fig. S1A,B). This was identical to the coordination environment in the wild-type protein structure^11^ (PDB entry 1N68), except that the additional water molecular served as a ligand to Cu8 in the G304K crystal structure.

In the G304K crystal structure, although the T1 and T3 Cu atoms are fully occupied, T2 Cu atom is depleted. Partial depletion of the four catalytic Cu atoms, especially the T2 Cu atom, was found in crystal structures of some CueO and other MCOs^17–20^. Analyses of X-ray data collection showed that X-ray radiation can lead to decrease in metal occupancy, even depletion^21–24^. Notably, the estimated absorbed dose during data collection of G304K crystal structure was 8.9 MGy using *RADDOSE-3D*^25^. For CueO, the removal of the T2 Cu atom, and even the loss of all four Cu atoms in the apo-CueO (PDB entry 3NSF), did not affect the overall fold^15,20^.

We tried to find a possible Cu-binding site (substrate Cu (sCu)^11^, first found in the PDB entry 1N68) located on the MR helix, like the one that exists in the wild-type enzyme structure. When we examined the mutant protein crystal, we found no evidence of a Cu ion at the corresponding site in the structure. Even with excess Cu ions, no electron density that could be assigned as Cu was present on the MR helix of G304K.

To exclude the possibility that the Cu content was affected in the preparation or purification process of CueO, we measured the total Cu content in the CueO using the ICP-MS (inductively coupled plasma mass spectrometry) method: the total Cu content in the purified wild-type CueO was 5.4 per protein molecule, and that in the G304K 5.1 per protein molecule (the experimental error in determining the Cu content was ca 10%). The result implied that four intrinsically catalytic Cu atoms were incorporated into the active site in the G304K. To see whether or not T2 Cu active site was occupied by Cu atoms within CueO in solution, we performed the electron paramagnetic resonance (EPR) spectrum experiment. Both signals resulting from type I and type II Cu atoms were observed in the EPR spectrum of the wild-type CueO^13,26^ (Fig. S2). An EPR signal characteristic of T2 Cu atom was apparent in the G304K, similar to that in the wild-type CueO (Fig. S2). These results suggested that T2 Cu active site was apparently occupied in the G304K in solution.

### Structural comparison of G304K in the presence and absence of excess Cu ions reveals Cu-induced conformational changes

**Figure 2:**
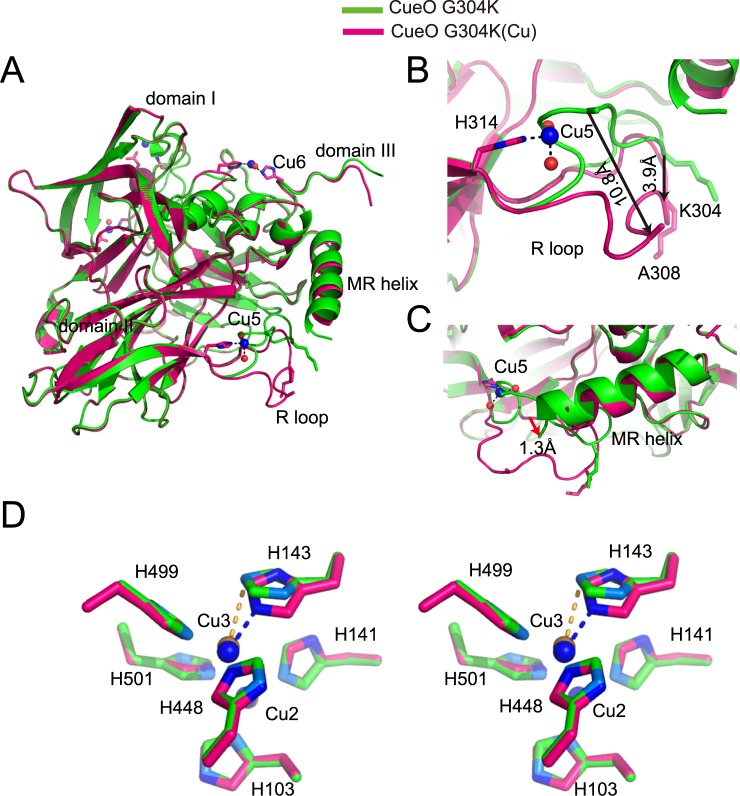
Structural comparison between CueO G304K mutant in the absence and presence of excess Cu ions. (**A**) Overall structural comparison of G304K mutant in the native state (in the absence of Cu ions) (green) and the Cu-bound state (in the presence of Cu ions) (hotpink). (**B**) Locally enlarged view of two different conformations of R-loop. Upon binding of Cu ions, the R-loop swings away. The Cα atoms of residues A308 and K304 of R-loop move a distance of approximately 10.8 Å and 3.9 Å, respectively, when binding of Cu ions, as black arrows indicated. The two residues are shown as sticks. (**C**) Close-up view of MR helices of G304K mutant in the presence of Cu ions (hotpink) superimposed on the one in the native state (green). The C-terminus of MR helix shifts a distance of approximately 1.3 Å upon binding of Cu ions, as a red arrow indicated. (**D**) The dinuclear type 3 Cu sites of G304K mutant in the presence of Cu ions (hotpink) superimposed on those in the native state (green) in stereo. Cu3 is ligated to residues H143, H448, and H499; whereas Cu2 is ligated to residues H103, H141, and H501. These are identical to those in the wild-type structure. Residues are shown as green and hotpink sticks, and Cu atoms are indicated with copper and blue spheres, in the native and Cu-soaked crystal of G304K, respectively. For clarity, only the ligations between Cu atoms and H143 are shown in yellow and blue dash in the native and Cu-soaked crystals, respectively.

The binding of metal ions to proteins can induce conformational change^27^. To determine whether the Cu ions affect the conformation of G304K, we performed the structural alignment analysis in which we compared G304K in the presence and absence of the excess Cu ions. We found that the two structures are essentially identical, but there were several important structural differences (Fig. 2A). First, in the structure transition from the native state (in the absence of Cu ions) to the Cu-bound state (in the presence of Cu ions), the R-loop dramatically sways, but exhibits significant movements to varying degrees at different locations (Fig. 2B). For example, the distance of Cα atom of Ala308 on the R-loop is 10.8 Å, while that of Cα atom of Lys304 becomes 3.9 Å. Moreover, the carboxyl-terminal of MR helix moves a distance of approximately 1.3 Å upon the binding of Cu (Fig. 2C).

For G304K structure in native state, the occupancy for one of T3 Cu centre (Cu3) was as low as 20%, suggesting that it was almost depleted. Upon the addition of excess Cu ions, the occupancy for Cu3 increased to 100%, i.e., it became fully occupied. Structural comparison between two states of G304K also revealed that the imidazole group of residue His143, which coordinates Cu3 along with His448 and His499, is flipped approximately 180° around the aromatic ring-C_β_ ligation of His143 to confer full occupancy for Cu3 upon the addition of the excess Cu ions (Fig. 2D). In contrast, the aromatic rings of other residues ligating Cu2 and Cu3 did not differ between the two states.

Taken together, these structural observations in the mutant crystal with and without excess Cu ions, suggested that Cu ions are responsible for the significant conformational changes around some regions, including the R-loop, MR helix and one of T3 Cu centre (Cu3). The addition of excess Cu ions result in Cu5 and Cu6, which cause the substantial conformational changes, in the R-loop, smaller conformational changes in the MR helix; and, more importantly, stabilize local structures.

### Structural comparison between G304K and the wild-type CueO in the presence of excess Cu ions

The comparison of the structures of G304K and wild-type CueO in the presence of excess Cu ions revealed that the mutant enzyme undergone a domain arrangement. We superimposed the G304K structure in the presence of excess Cu ions onto the structure of wild-type protein, the root mean square deviation (r.m.s.d.) was 1.593 Å for all main chain atoms (residues 31–516). When each domain was superimposed individually using Cα atoms, the r.m.s.d. values were 0.931 Å, 2.224 Å, and 1.017 Å for domains I, II and III, respectively, These results indicated that domain II of the mutant enzyme have undergone a significant internal rearrangement.

**Figure 3:**
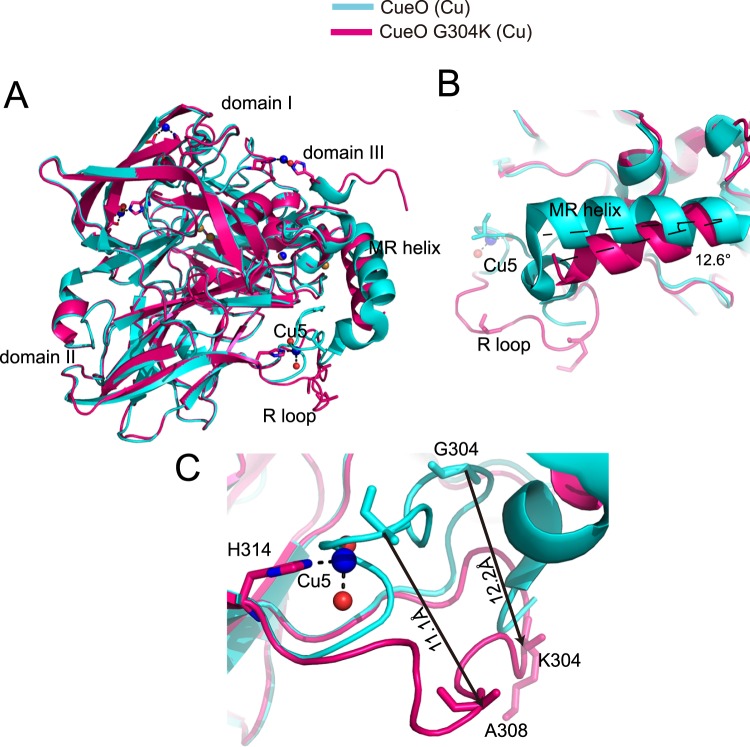
Structural comparison between CueO G304K mutant and wild-type enzyme in the presence of excess Cu ions. (**A**) Overall structural comparison of G304K mutant (hotpink) and wild-type (cyan) in the presence of Cu ions (PDB entry1N68). (**B**) Close-up view of the MR helices of G304K mutant (hotpink) in the presence of Cu ions superimposed on wild-type (cyan) in the same condition. The axis of MR helix of G304K rotate a degree of approximately 12.6° compared with that of wild-type, as an angle composed of two broken lines indicated. (**C**) Locally enlarged view of two different conformations of R-loop in G304K mutant (hotpink) and wild-type (cyan). The R-loop swings away. The Cα atoms of residues A308 and K304 (Gly304 in wild-type) of R-loop move a distance of approximately 11.1 Å and 12.2 Å, respectively, as black arrows indicated. The two residues are shown as sticks.

The structural alignment of the G304K mutant and wild-type protein in the presence of excess Cu ions highlighted dramatic conformational changes in the R-loop between sheet β9 and sheet β10 of domain II, and in the MR helix of domain III (Fig. 3A–C). In the G304K mutant structure in the presence of excess Cu ions, MR helix swayed as much as 12.6° away from the T1 Cu atom compared with its placement in the structure of wild-type CueO (Fig. 3B), and the R-loop also swayed away the MR helix (Fig. 3C). Noticeably, compared with the N-terminal part of the R-loop of the wild-type CueO (with or without excess Cu ions), the N-terminal part (residues 299–307) of the R-loop in the G304K mutant crystal structure significantly swayed in the absence of Cu ions (Fig. S3A,B). In contrast, the placement of the C-terminal part (residues 308–314) of the R-loop was almost not changed (Figs S3–S5). More importantly, the MR helix in the G304K structure swayed to a lesser degree in the absence of excess Cu ions than in the presence of excess Cu ions (Fig. S3C).

### Mechanism insights into how G304K mutation affects the catalytic activity of the enzyme

The above structural observations raised the question of how the conversion of a Gly residue into a Lys residue on the R-loop changed catalytic activity of CueO.

**Figure 4:**
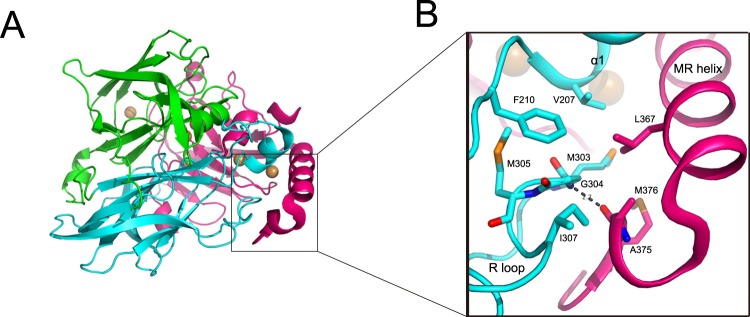
Interface between regulatory loop (R-loop) and methionine-rich helix (MR helix) in wild-type CueO. (**A**) Overall structure of wild-type CueO in the presence of Cu ions (PDB entry 1N68). Domain I-III is colored green, cyan, and hotpink, respectively. (**B**) Close-up view showing interface between R-loop and MR helix. In wild-type CueO, hydrophobic interactions are formed among residues V207, F210, M303, M305, I307, L367, A375, and M376; hydrogen bond between G304 of R-loop and A375 of MR helix. Oxygen atoms are shown in red, nitrogen in blue, sulfur in yellow.

In the wild-type structure^11^ (PDB entry 1N68), the R-loop of domain II is trapped in the mainly hydrophobic pocket, which is formed by the MR helix of domain III and helix α1 of domain II via hydrophilic and hydrophobic interactions (Fig. 4A). Hydrophobic interactions mainly occur among Leu367, Aal375, Met376 of the MR helix in domain III, Met303, Met305, and Ile307 on the R-loop in domain II, and Val207 and Phe210 of helix α1 and the neighboring loop of domain II (Fig. 4B). There is a hydrogen bond between the main chain amide of Gly304 on the R-loop in domain II and the main chain carbonyl group of Ala375 of the MR helix in domain III (Fig. 4B). Previous studies have reported that sCu is ligated by residues Met441, Met355, Asp439, and Asp360 on the MR helix^11^ (Fig. S1C). Therefore, the spatially stable arrangement of the MR helix promotes the formation of sCu in the wild-type protein structure.

In the G304K mutant structure, the R-loop is not trapped in the mainly hydrophobic pocket, but sways away the core domain of the enzyme. Because of the bulky side chain and positive charge of the Lys residue in G304K mutant, the R-loop could be significantly repelled out of the hydrophobic pocket that is mainly formed by the MR helix (Figs 2A and S3). With the addition of excess Cu ions, the R-loop sways more dramatically so that it is fully repelled out of the hydrophobic pocket (Figs 3A and S3).

**Figure 5:**
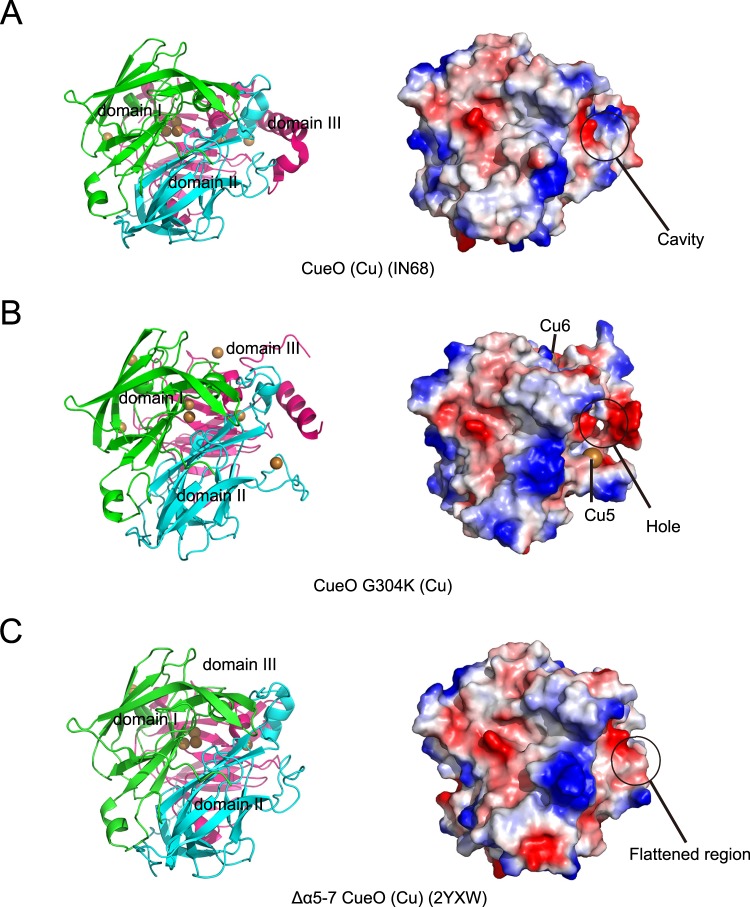
Structural comparison among different versions of CueO, including *E*. *coli* CueO (PDB entry 1N68) (**A**) G304K mutant (**B**) and Δα5–7 (PDB entry 2YXW) (**C**). The structures are represented in cartoon (left) and electrostatic surface (right). Domain I-III is colored green, palecyan, and magenta, respectively. In electrostatic surface, red present negative electrostatic potential; blue positive electrostatic potential. For the electrostatic surface presentation of G304K mutant, Cu5 and Cu6 are shown in copper spheres.

The most obvious feature in the CueO structure that is not found in the related enzymes ascorbate oxidase and laccase, is the methionine-rich region consisting of the MR helix and the following unresolved loop in the most CueO structures^10,11,15^. The MR helix is thought to block access to the T1 Cu site. A narrow cavity is formed between the MR helix and the core domain of the enzyme to reluctantly allow access of the organic substrate to the T1 Cu site (Fig. 5A). However, Δα5–7 CueO^13^, a truncated version of CueO that lacks the MR methionine-rich region, showed notably enhanced activities for substrates, because the region that hinders the access of substrates to the T1 Cu site is flattened (Fig. 5C). Interestingly, in G304K in the presence of excess Cu ions, the hole formed by the hydrophobic pocket without trapping the R-loop, was large enough to allow the passage of bulky organic substrates (Fig. 5B). Although the precise passageway through hole to T1 Cu site has not characterized, it is predicted that G304K will show markedly higher laccase activities for organic substrates, compared with that of the wild-type. Noticeably, in G304K in the absence of Cu ions, the hole formed by the hydrophobic pocket without fully trapping the R-loop was still open and allow the passage of organic substrates (Fig. S4A,B).

### Catalytic properties of mutants derived from G304K

**Figure 6:**
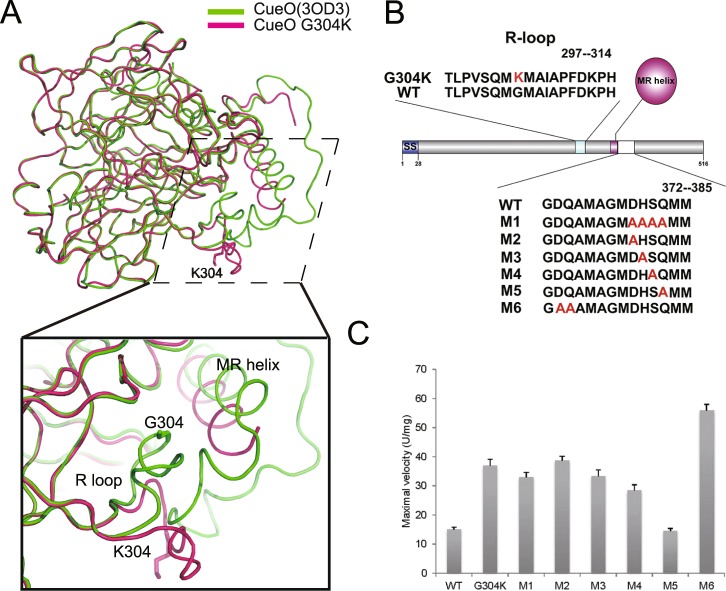
Analysis of laccase activity in six double mutants derived from CueO G304K mutant evidences that the R-loop modulates the enzyme activity. (**A**) G304K mutant structure superimposed on complete structure of wild-type protein (PDB entry 3OD3). Top shows superimposed overall structures; Bottom highlights relationships among R-loop, MR helix and disordered loop in G304K and wild-type enzyme. Residues are shown as sticks. (**B**) Diagram of several motifs and mutation points in CueO proteins. SS indicates signal sequence. R-loop is regulatory loop. Mutated residues in different mutants are shown in red. MR helix refers to methionine-rich helix. (**C**) Maximal velocities showing laccase activity of mutants shown in (**B**) and wild-type enzyme. Maximal velocities were measured using ABTS as substrate at pH4.5 and 37 °C. Data are mean ± standard deviation from at least three experiments.

In most of the reported CueO structures^10,11^, the loop following the MR helix has no electron density. This implied that the region is disordered and that it is the most flexible part of CueO structure. Only in the complete structure of CueO at a resolution of 1.1 Å (PDB entry 3OD3) is the previously unseen loop fully resolved^15^. Comparative analysis of the G304K structure with the complete CueO structure indicated that the resolved loop following the MR helix in the complete CueO structure is in close proximity to the dramatically wiggled R-loop in the CueO mutant structure (Fig. 6A). In the light of that finding, together with the above structural insights, we hypothesized that the hydrogen bonding and hydrophobic force may mediate the specific interaction between the R-loop and the disordered loop following the MR helix in G304K structure, resulting in the dramatic movement of the MR helix.

On the basis of this rationale, we cloned and purified six double mutants (including M1-M6) based on G304K mutant and measured the laccase activities of these mutants (Fig. 6B). The steady-state laccase activities of the wild-type CueO and mutants were measured using ABTS as the substrate. The resulting *Km* and *Vmax* values are listed in Table 2. The G304K mutant enzyme showed much higher catalytic activity than that of the wild-type, consistent with our previous findings^16^ (Fig. 6C). M1, which contained consecutive four point mutations, had lower catalytic activity than that of G304K. Intriguingly, among the four double mutants M2-M5, M5 had lower activity than that of G304K, but similar activity to that of the wild-type. This suggested that Gln383 plays a critical role in enhancing the interaction between the R-loop and the disorder region possibly by making hydrogen bond with Lys304 (Fig. 6C). Unexpectedly, M6 showed substantially higher laccase activity than those of the wild-type and G304K (Fig. 6C). It is speculated that in M6, not only the hydrogen force between Lys304 and Gln383, but also the hydrophobic interaction between consecutive mutated alanine residues and the hydrophobic residues on the wiggled R-loop, such as Met303, Met305, and Ile307, may reinforce the swing of MR helix. Therefore, in M6, the hole around the access to T1 Cu site is expected to become wider to accommodate bulkier organic substrates, to enhance the catalytic activity.

**Table 2:** Kinetic parameters of the wild-type CueO and various CueO mutants for ABTS oxidase activity.

	*K*_m_ (mM)	*V*_max_ (U mg^−1^)^a^
WT	3.79 ± 0.50	15.13 ± 0.74
G304K	1.88 ± 0.40	37.00 ± 2.17
M1	2.25 ± 0.41	32.94 ± 1.72
M2	2.40 ± 0.31	38.77 ± 1.47
M3	2.40 ± 0.53	33.36 ± 2.18
M4	2.46 ± 0.53	28.47 ± 1.87
M5	2.55 ± 0.53	14.54 ± 0.92
M6	1.72 ± 0.23	56.00 ± 1.99

^a^One unit is the amount of enzyme that oxidizes 1 μmol of ABTS per minute in 0.1 M citrate/phosphate buffer (pH 4.5) at 37 °C.

To observe the effects of Cu-induced conformational changes in the G304K structure, we added Cu (II) ions and then determined the laccase activities of G304K and important mutants derived from it, including M5 and M6. The addition of Cu led to great increases in the laccase activity for G304K than for the wild-type enzyme, implying that the mutant had better stronger Cu-binding ability than that of the wild-type CueO (Fig. S6). The assay results were consistent with the results of structure analysis, that is, that the addition of Cu makes the hole (which is formed by the hydrophobic pocket without trapping the R-loop) large enough to allow the passage of bulky organic substrates. The addition of Cu similarly increased the laccase activity of M5, M6 and G304K (Fig. S6).

The wild-type CueO is an excellent cuprous oxidase^12^. We conducted the cuprous oxidase activity assay for the wild-type CueO and important mutants derived from it including CueO G304K, M5 and M6. The cuprous oxidase activity of G304K mutant (*Vmax* = 6.3 U/mg) was about 20% that of the wild-type CueO (*Vmax* = 29.5 U/mg), while the *Km* value for G304K mutant (*Km* = 80.3 uM) was four times lower than that of the wild-type CueO (*Km* = 427.8 uM) (Fig. S7). The kinetic parameters determined for the cuprous oxidase activity of M5 and M6 were similar to those of G304K (Fig. S7). Thus, the cuprous oxidase activities of these important CueO mutants were also inhibited.

## Discussion

Methionine-rich regions are found in the sequence of numerous proteins involved in Cu homeostasis, such as CueO^10^, PcoA^28^. The methionine-rich region in the CueO lies over the T1 Cu site, so it can interfere with the access of substrate to T1 Cu site^10^. This region harbours multi-Cu-binding sites to confer its cuprous oxidase activity^29^. The loss of the complete methionine-rich region do not change the overall structure, but significantly affects the enzymatic activity^13,14^, especially laccase activity, suggesting that the methionine-rich region is very important for activity. However, how the enzyme activity is regulated via methionine-rich region is not fully understood.

In our previous work, we isolated and characterized the *E*. *coli* CueO mutants from the sludge in a chemical plant, among which G304K had 2.7-folds higher for activity than that of the wild-type^16^ (Fig. 6C). The finding is surprising, because in CueO structure, Gly304 located on the R-loop was 14 Å away from the T1 Cu site, and mutation adjoining the catalytic sites in the other point mutants such as M441L generally results in the loss of enzyme activity^11^.

To determine the reason for the change of enzyme activity, we analysed the crystal structure of G304K. Compared with the wild-type enzyme structure, G304K showed dramatic conformational changes in the MR helix and R-loop (Fig. 3). Interestingly, binding of Cu ions induces the local conformational changes, especially the sway of the R-loop (Fig. 2). Obviously, the sway of the R-loop triggered significant movements of the methionine-rich region, resulting in the appearance of a hole through which the substrate could access to T1 Cu site (Fig. 5B). The structural changes may explain the increased laccase activity of the mutant enzyme.

Functional analyses of CueO double mutant confirmed the interaction between the R-loop and the methionine-rich region. In addition, cuprous oxidase activity was inhibited in the CueO mutants G304K, M5 and M6. Although the coordinating residues for sCu were still intact in the CueO mutant structure, the coordinating environment for sCu had changed and no electron density which could be assigned as Cu was detected at the sCu-binding site. The conformational change resulted from the conversion of Gly residue into Lys residue on the R-loop, which may have affected the cuprous oxidase activity of the CueO mutant. Collectively, our results define how the R-loop regulates the enzyme activity by modulating the methionine-rich region.

In Cu-binding metaloenzymes, amino acid residues coordinating Cu atoms in the proteins include His, Met, Cys, Asp and Glu^1,30^; however, histidine ligation with Cu is by far the most frequent^1,31^. We and others^11,13^ have found some novel Cu-binding sites on the surface of the CueO crystal structure, apart from the essential catalytic Cu atoms buried in the core domains. In our research, only His was found to coordinate with Cu5 and Cu6, whereas His, Asp and Glu were found to coordinate with Cu7 and Cu8 (Figs 1 and S1A,B), as has been reported for the previously described structures of CueO^11,13^ (PDB entries 1N68 and 2YXW).

Our results raised the interesting question as to the role of Cu-binding sites on the surface of the wild-type CueO and G304K mutant enzyme. On the basis of the results of our study and previous studies, we suggest that Cu-binding sites on the surface of CueO have at least two practical roles: to allow for the transfer of Cu ions among Cu-binding proteins; and to induce a conformational change in the CueO structure. Previous studies have shown that CueO is localized in the periplasm of *E*. *coli* cell, along with the other Cu-binding proteins such as bacterial MCO PcoA^28^ and its putative partner PcoC^32^. Moreover, it is generally thought that the Cu atoms in the Cu-binding proteins are well poised for metal transfer because of their low coordination number and their solvent accessibility^33^. The coordination number Cu5, Cu6 and Cu8, which are bound to the surface of CueO mutant, was 3, whereas it was as low as 2 for Cu7 (Figs 1 and S1A,B). This suggests that Cu atoms may be easily transferred among the Cu-binding proteins, including CueO, PcoA, and PcoC, within the periplasm of bacteria cells. Further research is required to explore the implications and mechanism of this transfer. The second possible role of surface Cu-binding sites is to change the conformation of the CueO structure. The structural comparison of G304K with and without excess Cu ions showed that with the excess Cu ions, Cu5, Cu6 and the other Cu atoms occupied the sites on the surface, the R-loop displaed dramatic movements, and the imidazole group of His143 rotate 180° upon incorporation of Cu atom into Cu3 position (Fig. 2). To our knowledge, it is the first time that Cu atom have been shown to change CueO structure. Our analyses showed that the laccase activity of the CueO mutants was dependent on Cu, suggesting that it is mainly Cu5 occupancy that makes the hole large enough to allow the passage of bulky organic substrates to enhance laccase activity.

In summary, analyses of X-ray crystal structures of G304K revealed that the R-loop, without being trapped in the pocket, regulates the catalytic activity of CueO by giving rise to a local conformational change around the MR-helix. Extensive engineering of the protein based on the R-loop and the unstructured methionine-rich region are needed to generate substrate-specific CueO mutant for potential applications in biotechnology.

## Methods

### Cloning, Expression and Purification

Using the standard PCR cloning strategy, the truncated CueO G304K gene (amino acids 29–516) lacking the signal sequence-encoding region from the metagenome was cloned into the *Nde*I and *Xho*I sites of modified pET28a (+TEV) vector. The construct was confirmed by DNA sequencing.

The recombination CueO G304K gene was overexpressed as 6× His-fusion protein in *Escherichia coli* strain Rosetta2 (DE3) pLysS (Novagen). *E*. *coli* cultures harboring fusion protein were incubated in the shaker at 37 °C at 220 rpm. Subsequently, the shaker temperature was shifted to 25 °C, when its density reached an OD_600nm_ of 0.8. The expression of recombination proteins was induced with the supplement of isopropylb-D-thiogalactoside (IPTG) 1.0 mM plus CuCl_2_ 0.25 mM final concentration, respectively. After growth for further 1 h at 25 °C at 160 rpm, the cultures were kept at static incubation until harvest. The cell sediments were suspended in lysis buffer containing 20 mM Tris-HCl pH 8.0, 20 mM imidazole and 500 mM NaCl, and disrupted in a French press at 12,000 psi for three cycles. To remove cell debris, the lysate was centrifuged for 40 min at 13,000 × g. The supernatant were collected and loaded on the equilibrated Ni^2+^ Chelating Sepharose^TM^ Fast Flow (GE Healthcare). After several gradually crush wash, CueO G304K protein was eluted with a buffer containing 20 mM Tris-HCl pH 8.0 and 500 mM NaCl supplemented with 300 mM imidazole. The eluate was digested using TEV protease to remove 6× His tag, while dialyzed against 20 mM Tris-HCl, 150 mM NaCl, pH 7.5, overnight at 4 °C. The eluate was filtered using a 0.45 μm filter and concentrated prior to loading onto a 25 ml superdex^TM^ 200 10/300 GL (GE Healthcare). The peak fraction was pooled and concentrated to ~15 mg/ml for crystallization.

The other CueO mutants were purified according to the same protocol as for CueO G304K protein. Purified CueO protein and derived mutants were analyzed by SDS-PAGE for purity.

### Crystallization and X-ray Data Collection

CueO G304K protein was crystallized by the sitting-drop vapor diffusion method at 22 °C, with 0.7 μl 15 mg/ml protein solution mixed with an equal volume of reservoir solution. Initial crystallization trials screen needle-shaped crystals in a mother liquor containing 0.2 M potassium formate (pH 7.0), 20% (w/v) Polyethyleneglycerol 3,350. Further optimization of the crystallization condition led to the appearance of rod-shaped crystals in the well in which 0.7 μl 10 mg/ml protein solution was mixed with an equal volume of reservoir solution containing 0.2 M potassium formate (pH 8.5), 20% (w/v) Polyethyleneglycerol 3,350. Crystals were transferred to 30% Polyethyleneglycerol 3,350 prior to flash-frozen in liquid nitrogen. For Cu-protein complex, crystals were soaked in the identical solution plus 10 mM CuCl_2_ for 60 min, and transferred to 30% Polyethyleneglycerol 3,350, and flash-frozen in liquid nitrogen.

The data set of CueO G304K protein was collected at beamline BL17U of the Shanghai Synchrotron Radiation Facility (SSRF). The data set of CuCl_2_-soaked protein was collected at beamline BL1A of Photon Factory (PF) at the wavelength of 1.1 Å, and the anomalous data was collected at the Cu absorption edge of the wavelength of 1.37 Å at beamline BL5A of PF. The data were indexed, integrated and scaled with HKL2000^34^.

### Structure determination and refinement

The initial phases determined by molecular replacement using the program Phaser^35^ from CCP4 program suit^36^, with the crystal structure of full length *E*. *coli* CueO (PDB entry 1N68) as a searching model.

The structure refinement was carried out with Phenix^37^ and Refmac^38^. Model building was carried out by iterative rounds of manual building with COOT^39^. According to the anomalous difference Fourier maps, the locations of Cu ions were identified. MolProbity^40^ was used to validate the structure. Data collection and refinement statistics are listed in Table 1.

All structural figures were rendered using PyMOL programme (http://www.pymol.sourceforge.net/). For comparisons, the structures were superposed using the program LSQKAB of the CCP4 suite^36^.

### Absorbed dose calculation

Absorbed dose calculations were performed using *RADDOSE-3D*^25,41^. The values of the beam parameters (including the energy, flux, profile, spot size, etc), crystal properties (unit cell, space group, number of molecules per asymmetric unit, composition, size and thickness of the crystal), and wedge parameters (angle of the rotation, exposure time, the angular step size) were provided as the input information. Among the values, the beam energy was 12.1KeV, the beam flux was 4.1 × 10^12^ photons per second, and exposure time was 36 second^25^.

### The copper content and electron paramagnetic resonance spectrum

The concentration of protein in enzyme pools was routinely determined by the method of Bradford using bovine serum albumin as the standard. Copper content was determined by X-series ICP-MS (inductively coupled plasma mass spectrometry) (ThermoElemental, Beverly, Mass.) operated by the Key Laboratory for Biomedical Effects of Nanomaterial and Nanosafety of Institute of High Energy Physics from CAS. X-band electron paramagnetic resonance (EPR) spectra were recorded on a BrukerESP 300 spectrometer (Billerica, Mass.) operated by State Key Laboratory of Natural and Biomimetic Drugs of Peking University.

### Mutagenesis of CueO

Site-directed mutagenesis of CueO was performed as previously described^42^. Briefly, pET30-CueO_G304K was used as a template. Desired mutations of CueO_G304K were amplified by complementary mutagenic primers (M1F/R-M6F/R) (S Table 2) with FastPfu DNA polymerase (TIANGEN, Beijing, China). The PCR product was treated with *Dpn* I to remove the parental template DNA and introduced into *E*. *coli* DH5α. In this way, the pET-CueO_G304K derived plasmids pET-M1, pET-M2, pET-M3, pET-M4, pET-M5 and pET-M6 with specific mutations (S Table 1), verifying by DNA sequencing, were obtained. All these plasmids were transformed into *E*. *coli* BL21(DE3) for protein expression.

### Laccase activity assay

The substrate ABTS [2,2′-azino-bis (3-ethylbenzthiazoline-6-sulfonic acid)] (sigma) was used to assess the laccase activity of the wildtype CueO and various CueO mutants by monitoring the change in absorption at 420 nm as described previously^16^. The assay mixture was consisted of 100 mM citrate/phosphate (pH 4.5) or 100 mM sodium acetate (pH 4.5), 2 mM ABTS, specified or varied concentration of CuCl_2_ at 37 °C. To initiate the reaction, purified enzymes were added at the final concentration 3 nM.

One unit of enzyme activity was defined as the amount of the laccase that oxidized 1 μmol of ABTS substrate per min. Activity measurements were plotted as a function of substrate using GraphPad 5.0 (La Jolla, CA) and kinetic constants, *Km* and *Vmax*, were evaluated using a leastsquares fit of a Michaelis-Menten curve.

### Cuprous oxidase activity assay

The cuprous oxidase activities of the wild-type CueO and various CueO mutants were measured indirectly by monitoring oxygen consumption using a Clark-type oxygen sensor electrode (Hansatech, Cambridge, UK), with [Cu(I)(CH3CN)4]PF6 (Sigma) as a substrate, as described by Singh *et al*.^12^.

In brief, stock solutions of the substrate were freshly prepared in degassed and nitrogen-purged acetonitrile. To initiate the reaction, a 100 ul aliquot of Cu(I) substratewith varied concentration, was added to an air-saturated mixture containing 312 nM enzyme, 100 mM Tris-acetate (pH5.0), 5% acetonitrile, and 0.2 mM Cu(II). All kinetic measurements were made at 18 °C and pH 5.0. Enzyme velocities were calculated after subtracting the aerial oxidation of Cu(I) in the absence of enzyme. Activity measurements were plotted as a function of substrate using GraphPad 5.0 (La Jolla, CA) and kinetic constants, *Km* and *Vmax*, were evaluated using a least squares fit of a Michaelis-Menten curve.

### Accession numbers

The atomic coordinates and structure factors have been deposited in the Protein Data Bank (http://wwpdb.org/) with accession codes 5YS1 (CueO G304K mutant) and 5YS5 (Cu-soaked CueO G304K mutant).

## Electronic supplementary material

Supplementary information
