# Supplementary material for: Crystal structures of multicopper oxidase CueO G304K mutant: structural basis of the increased laccase activity
Source: Sci Rep. 2018 Sep 24;8:14252. doi: 10.1038/s41598-018-32446-7 (PMC6155172; doi:10.1038/s41598-018-32446-7)
Supplement: Supplementary file 1 — Supplementary information [file 41598_2018_32446_MOESM1_ESM.pdf]

Crystal structures of Multicopper Oxidase CueO G304K mutant: structural basis of the increased laccase activity

Hanqian Wang, Xiaoqing Liu, Jintong Zhao, Qingxia Yue, Yuhua Yan, Zengqiang Gao, Yuhui Dong, Zhiyong Zhang, Yunliu Fan, Jian Tian, Ningfeng Wu, Yong Gong

Supplementary Information

Supplementary Figure legends

Figure S1. Several Cu-binding motifs in the reported CueO structures.

A. Residues H494 and E110, shown as sticks, coordinate Cu7 (blue sphere) in G304K mutant, as described in the legend to Fig. 1B.
B. Residues H488 and D132, shown as sticks, and one water molecular indicated with red sphere, coordinate Cu8 (blue sphere) in G304K mutant, as described in the legend to Fig. 1B.
C. Residues M358 and M362, shown as sticks, and one water molecular indicated with red sphere, coordinate one Cu. Residues M364, M368 and M376, shown as sticks, coordinate another Cu. Residues M355, D360, D439 and M441, shown as sticks, coordinate sCu.
D. Residues H145 and M417, shown as sticks, coordinate Cu.
In (C) and (D), which originate from in the structure of Cu-soaked C500S mutant (PDB entry 3NT0), Cu atoms are shown in copper, sulfur in limon, nitrogen in blue.

Figure. S2. Electron paramagnetic resonance (EPR） spectrum of the wild-type (green lines) and G304K mutant (red lines).

Absorbance bands from the type 1 (I) and type 2 (II) Cu centers are indicated. The inset depicts a five-fold amplification of the hyperfine region. Measurement conditions: 0.02 M Tris-HCl buffer (pH 7.5) at 95 K; microwave frequency, 9.44 GHz; microwave power, 7.0 mW; modulation frequency, 100 kHz; modulation amplitude, 5 Gauss; sweep width, 1200 Gauss; sweep time, 2 min.

Figure S3. Structural comparison among G304K mutant in the absence and presence of excess Cu ions and wild-type in the presence of excess Cu ions.

A. Overall structural comparison of G304K mutant in the native state (in the absence of Cu ions) (green), in the presence of Cu ions (hotpink) and the wild-type (cyan) in the presence of Cu ions (PDB entry1N68).
B. Locally enlarged view of three different conformations of the R loop from G304K mutant in the native state (green) and in the presence of Cu ions (hotpink), and the wild-type (cyan). The distance between Cα atom of residue

K304 of G304K mutant in the native state (green) and Cα atom of residue G304 of wild-type (cyan) is 8.7 Å.
C. Close-up view of the MR helices of wild-type (cyan) in the presence of Cu ions superimposed on G304K mutant in the presence of Cu ions (hotpink) and in the native state (green).

Figure S4. Structural presentation of G304K mutant in the absence of excess Cu ions.

The structural representation in cartoon (left) and electrostatic surface (right) was described in the legend to Fig. 5.

Figure S5. Superposition of structures of four different versions of CueO.

The different versions of CueO include wild-type in the presence of excess Cu ions (PDB entry 1N68), CueO harboring complete structure (PDB entry 3OD3), G304K mutant in the native state (in this study) and in the presence of excess Cu ions (in this study).
For simplification, Cu atoms do not display.

Figure S6 Cu(II) activation of the ABTS oxidase activities of the wild-type (circles), G304K mutant (squares), M5 (regular triangles) and M6 (inverted triangles).

Oxidation of ABTS, in the presence of $CuCl_2$ with varied concentrations, was measured in the reaction mixture containing 0.1 M sodium acetate (pH 4.5) and 2 mM ABTS at 37 °C.

Figure S7 Steady-state cuprous oxidase activity catalyzed by the wild-type (circles), G304K mutant (squares), M5 (regular triangles) and M6 (inverted triangles).

One unit is the amount of enzyme that oxidizes 1 μmol of substrate per minute in 0.1 M Tris-acetate buffer (pH 5) at 20 °C. Data are mean ± standard deviation for at least three measurements at each substrate concentration.

The curves are a nonlinear fit of the Michaelis-Menten equation through the plotted data, generating *Km* and *Vmax* values.

Supplementary Table S1. Primers used for construction of the site-directed mutants of CueO.

CueO mutant	Mutated amino acid			Sequence of the forward primer (5′ to 3′) [a]
	position	Wild type	Mutant	
M1F	380-383	DHSQ	AAAA	ATCAGGCGATGGCCGGGATGGCTGCCGCCGCGATGATGGGCCATATGGGGCA
M2F	380	D	A	ATCAGGCGATGGCCGGGATGGCTCACAGCCAGATGATGGGCCA
M3F	381	H	A	AGGCGATGGCCGGGATGGATGCCAGCCAGATGATGGGCCATAT
M4F	382	S	A	CGATGGCCGGGATGGATCACGCCCAGATGATGGGCCATATGGG
M5F	383	Q	A	TGGCCGGGATGGATCACAGCGCGATGATGGGCCATATGGGGCA
M6F	373-374	DQ	AA	TGCTAATGGAGAAATATGGCGCTGCGGCGATGGCCGGGATGGATCA

[a] The reverse primers completely complementary with these forward primers are not listed. Mutations are shown in shade.

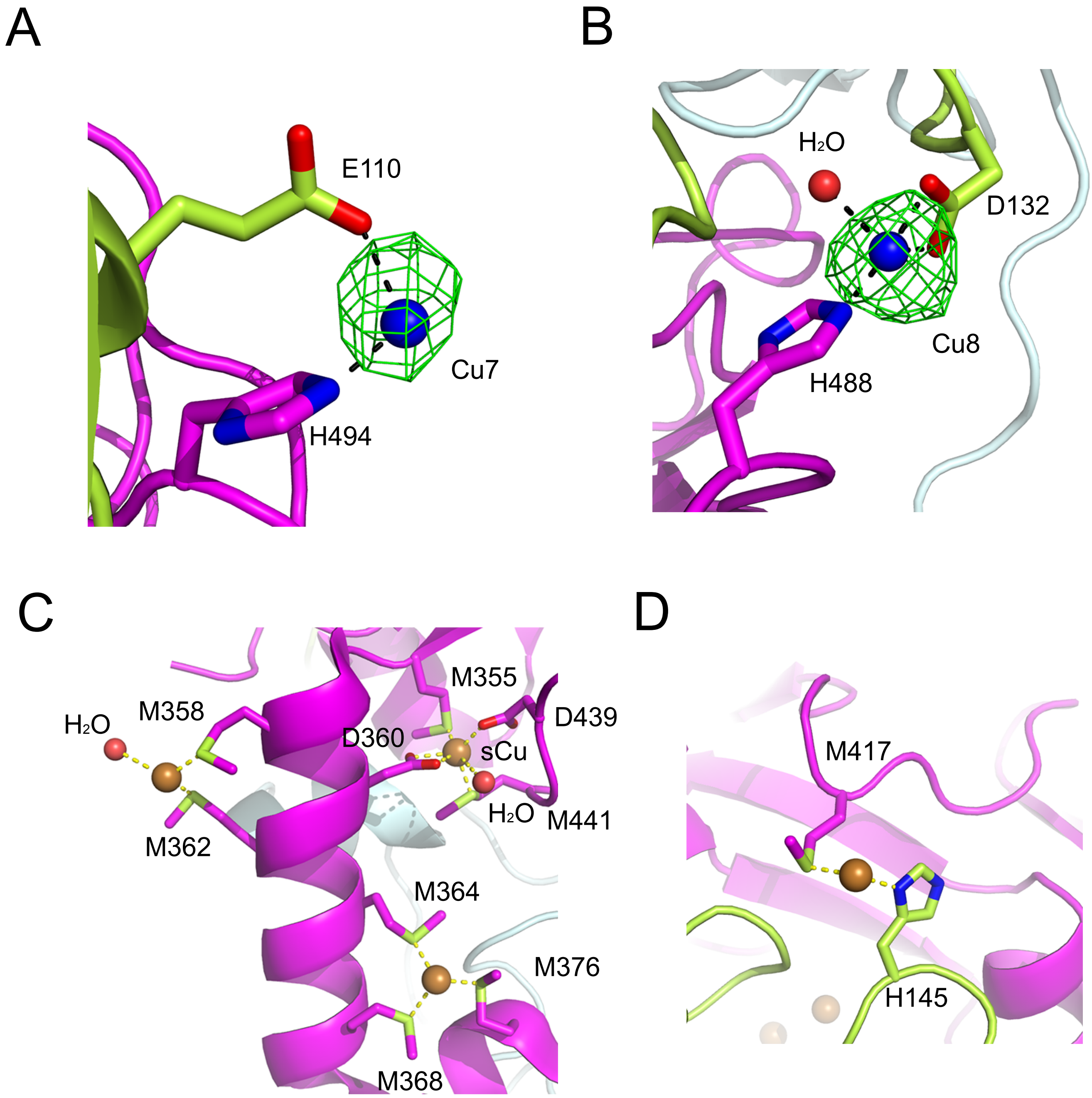
A
E110
Cu7
H494
B
H_2O
D132
Cu8
H488
C
H_2O
M358
M362
M355
D439
D360
sCu
H_2O
M441
M364
M376
M368
D
M417
H145

Figure S1

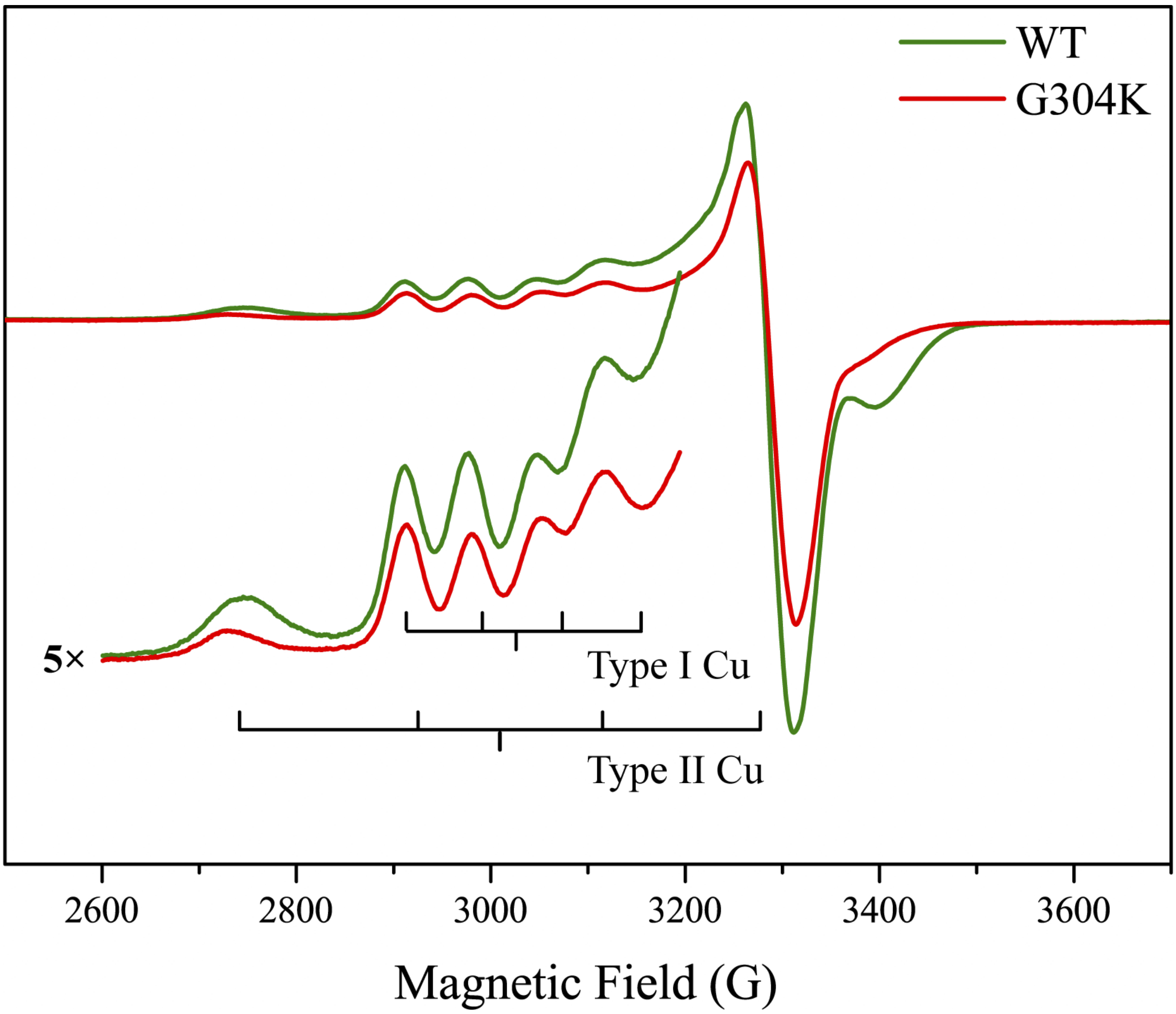
WT
G304K
5×
Type I Cu
Type II Cu
2600
2800
3000
3200
3400
3600
Magnetic Field (G)

Figure S2

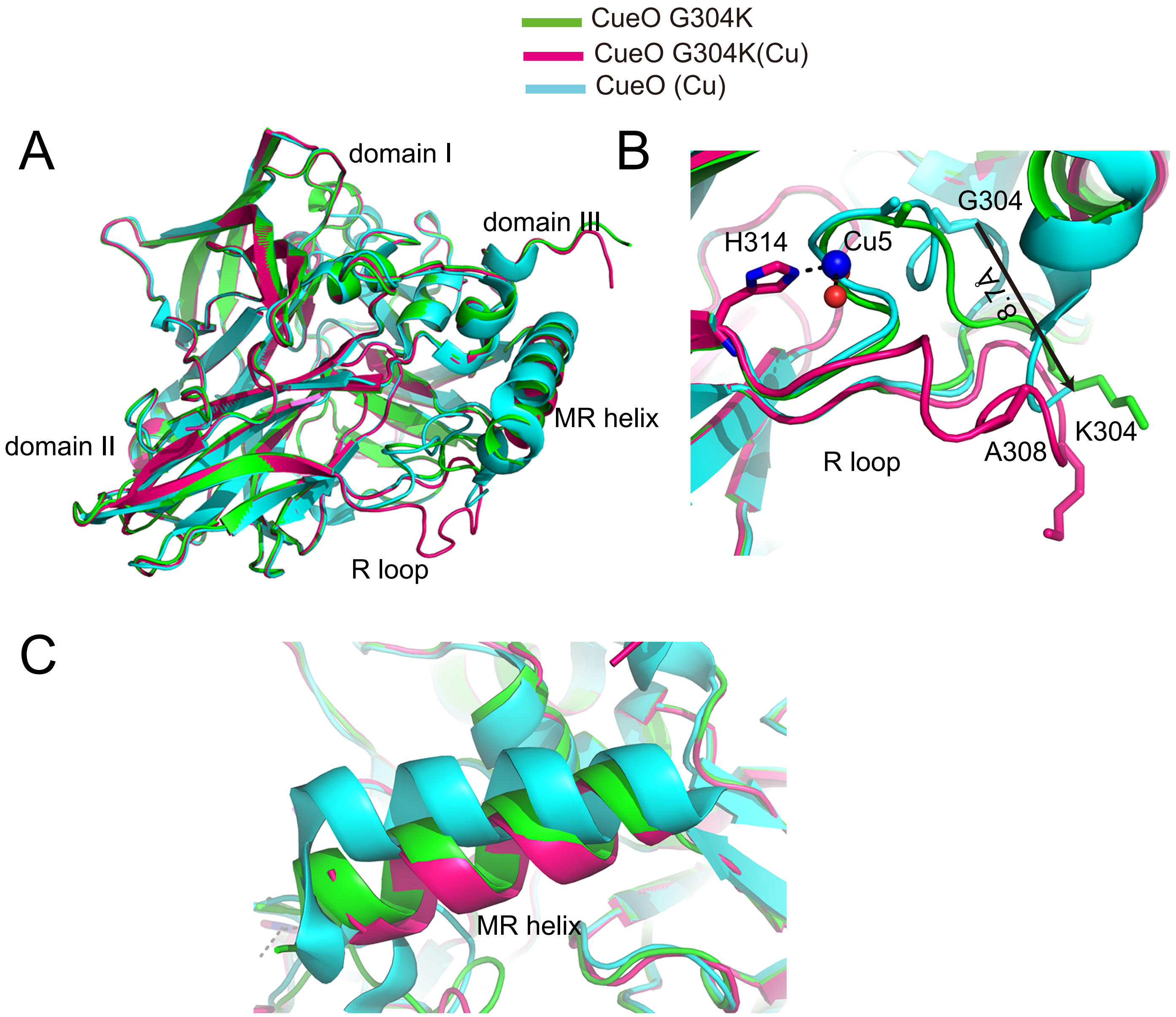
CueO G304K
CueO G304K(Cu)
CueO (Cu)
A
domain I
domain III
MR helix
domain II
R loop
B
H314
Cu5
G304
8.7Å
K304
A308
R loop
C
MR helix

Figure S3

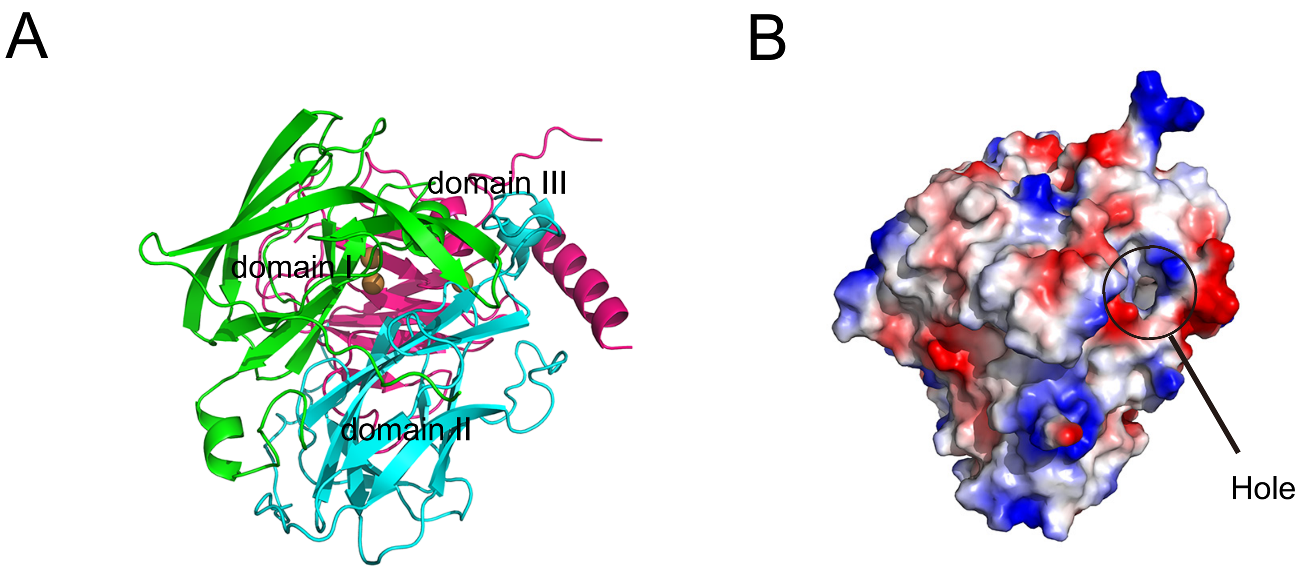

CueO G304K

Figure S4

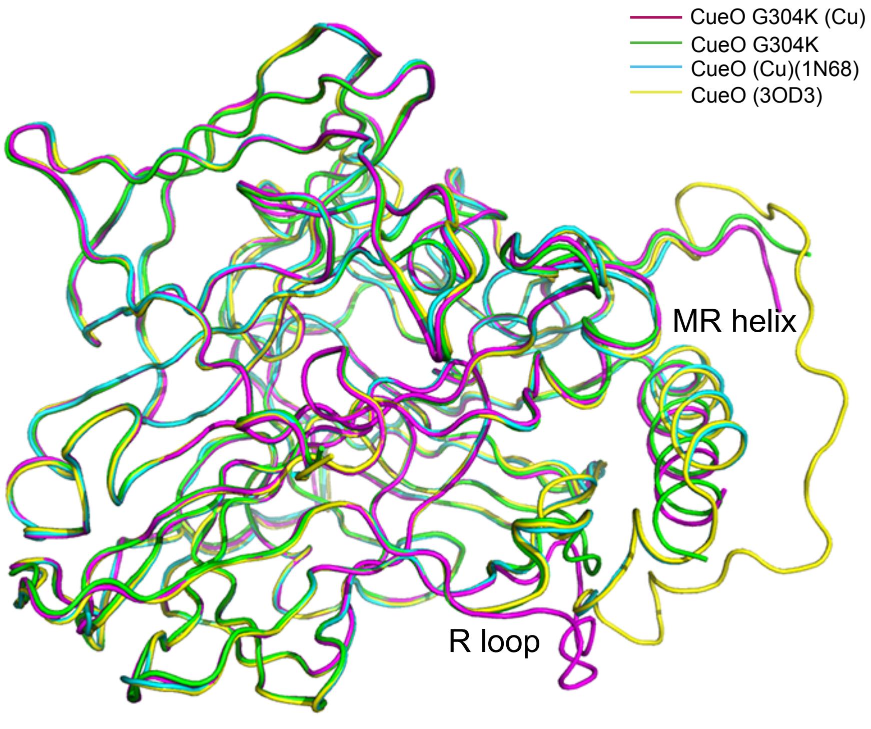
CueO G304K (Cu)
CueO G304K
CueO (Cu)(1N68)
CueO (3OD3)
MR helix
R loop

Figure S5

.

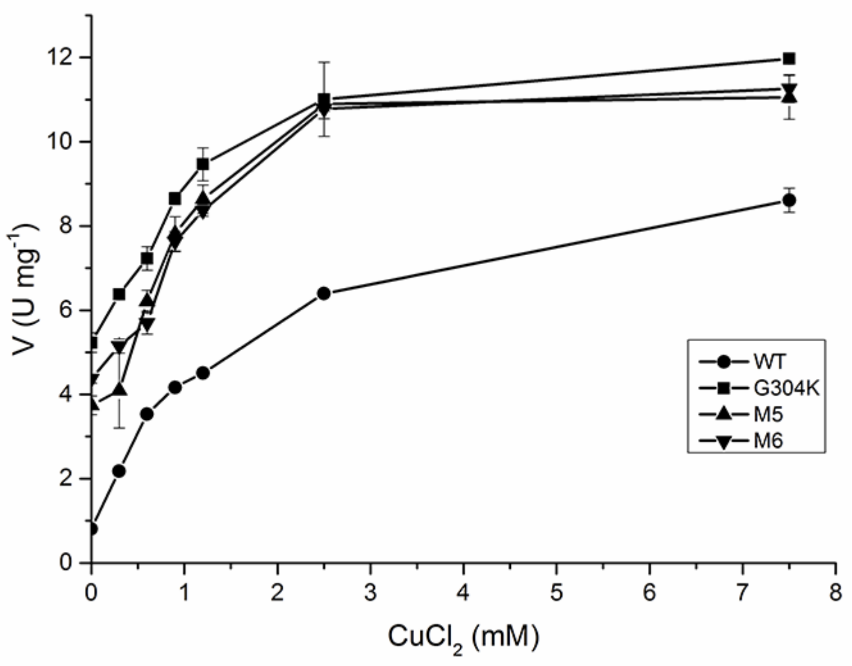

Figure S6

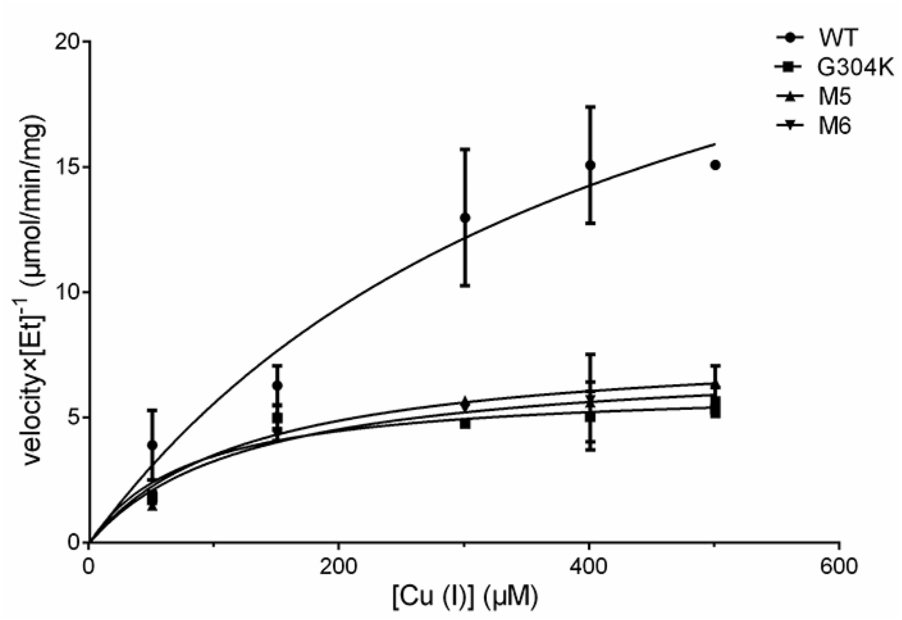

WT
G304K
M5
M6
velocity×[Et]$^{-1}$ (µmol/min/mg)
20
15
10
5
0
0
200
400
600
[Cu (I)] (µM)

Figure S7
